# Human Gene Control by Vital Oncogenes: Revisiting a Theoretical Model and Its Implications for Targeted Cancer Therapy

**DOI:** 10.3390/ijms13010316

**Published:** 2011-12-27

**Authors:** Rudolph E. Willis

**Affiliations:** Department of Medical Oncology, Cancer Treatment Centers of America, Eastern Regional Medical Center, 1331 Wyoming Ave, Philadelphia, PA 19124, USA; E-Mail: rudolph.willis@ctca-hope.com; Tel.: +1-215-537-7545; Fax: +1-215-537-7991

**Keywords:** oncogenes, gene regulation, gene transcription, transcription activator, targeted cancer therapy, signal transduction, carcinogenesis, protein kinase, cell cycle control, steroid hormone action

## Abstract

An important assumption of our current understanding of the mechanisms of carcinogenesis has been the belief that clarification of the cancer process would inevitably reveal some of the crucial mechanisms of normal human gene regulation. Since the momentous work of Bishop and Varmus, both the molecular and the biochemical processes underlying the events in the development of cancer have become increasingly clear. The identification of cellular signaling pathways and the role of protein kinases in the events leading to gene activation have been critical to our understanding not only of normal cellular gene control mechanisms, but also have clarified some of the important molecular and biochemical events occurring within a cancer cell. We now know that oncogenes are dysfunctional proto-oncogenes and that dysfunctional tumor suppressor genes contribute to the cancer process. Furthermore, Weinstein and others have hypothesized the phenomenon of oncogene addiction as a distinct characteristic of the malignant cell. It can be assumed that cancer cells, indeed, become dependent on such vital oncogenes. The products of these vital oncogenes, such as c-myc, may well be the Achilles heel by which targeted molecular therapy may lead to truly personalized cancer therapy. The remaining problem is the need to introduce relevant molecular diagnostic tests such as genome microarray analysis and proteomic methods, especially protein kinase identification arrays, for each individual patient. Genome wide association studies on cancers with gene analysis of single nucleotide and other mutations in functional proto-oncogenes will, hopefully, identify dysfunctional proto-oncogenes and allow the development of more specific targeted drugs directed against the protein products of these vital oncogenes. In 1984 Willis proposed a molecular and biochemical model for eukaryotic gene regulation suggesting how proto-oncogenes might function within the normal cell. That model predicted the existence of vital oncogenes and can now be used to hypothesize the biochemical and molecular mechanisms that drive the processes leading to disruption of the gene regulatory machinery, resulting in the transformation of normal cells into cancer.

## 1. Introduction

In the beginning, we could only speculate about the mysterious and seemingly incomprehensible processes within the human cell that govern gene regulation. Furthermore, an understanding of the deregulation of a cell’s normal gene regulation resulting in its transformation into a cancer was even more mysterious and challenging. We could only guess how a human gene is turned on or off. Our models of eukaryotic gene regulation consisted of vague diaphragms and marvelous, but nonspecific, terms that failed to reveal a unifying biochemical and molecular concept sufficient to explain how human genes are regulated, and how genomic deregulation becomes the pivotal event leading to carcinogenesis [1]. Although we now have an impressive understanding of the general processes involved in the transcription of eukaryotic cells [2], little did we realize that the beginning of our true understanding of such matters would come from a series of seemingly unrelated crucial discoveries.

Peyton Rous discovered that he could induce tumor formation in chickens by cell-free extracts [3]. Rous sarcoma virus proved to be a retrovirus [4]. These retroviruses utilize their RNA genetic codes to generate complementary provirus DNA which subsequently is integrated into the host cellular DNA at random sites [5]. When new retroviral RNA is generated, it may contain additional sequences derived from the DNA of its host. This fused genetic product may contain host genomic sequences that are directly involved in normal host gene regulation. A retrovirus may choose to transmit these regulatory proto-oncogene sequences to other infected cells resulting in cellular neoplastic transformation. Bishop and Varmus concluded that the Rous sarcoma virus, in fact, had hijacked the cell’s regulatory proto-oncogene, converting its protein product into an oncoprotein [6]. Another crucial discovery was the realization that there was a tumor virus and protein kinase connection, and the fact that these oncoproteins contained tyrosine kinase activity [7,8]. Protein phosphorylation reactions play a significant role in a host of cellular processes [9–11]. Understanding the astounding fact that these oncogenic viruses achieved their neoplastic effect by capturing a critical host gene regulatory sequence with protein kinase ability, paved the way for new insights about not only normal eukaryotic gene regulation, but also presented a new and potentially profound way of looking at the origins of cancer [12].

An incomprehensible amount of research has been achieved since the presentation of these central discoveries and concepts. The regulation of transcription by phosphorylation should be undisputed [13]. Yet, the previously proposed important role of phosphatase reactions in human gene regulation can no longer be ignored [11,12,14–22]. We now understand the magnificent role of proto-oncogenes in normal cell growth and development [23]. There is an increasing understanding of the biochemical pathways leading from extracellular stimuli through the cell’s membrane and into the cell’s cytoplasm. This sometimes intricate signal transduction cascade inevitably must end within the nucleus, where vital oncogenes must function [24,25]. And we now know that the most common domain that is encoded by cancer genes is the protein kinase [26]. The efforts to clarify the normal mechanisms involved in cell cycle control have been equally fruitful in shedding light upon the dysfunctional events during the cell cycle of a cancer cell [27–29]. It is not surprising that there is indeed a connection between the cell cycle and cancer. It is the cell cycle process that leads to normal cell proliferation. An abandonment of such a normal process is the essence of the neoplastic process. For example, we will see how in particular the cancer process impacts the crucial regulatory machinery of the G1 phase of the cell cycle.

Weinstein has proposed an insightful theory about the peculiar characteristics of the dysfunctional genomic activities of the cancer cell [30,31]. He has suggested that cancer cells become addicted to an overactive oncogene or a selective signal transduction pathway leading to activation of this dependent dysfunctional proto-oncogene. The effect of some recently developed molecular targeted cancer therapies may give clinical support to this notion [32]. Such a phenomenon would have an even greater significance if we assume that this event involves vital oncogenes. Such oncogenes would be the critical oncogenes that the cancer cell has become dependent upon for its survival.

Demonstrating the mere existence of vital oncogenes is insufficient for our complete understanding of the neoplastic process. A significant obstacle still would remain. How would these vital oncogenes usurp the crucial normal gene regulatory mechanisms? As stated earlier, commandeering the normal cell cycle control mechanisms is mandatory. Yet, these vital oncogenes would have to accomplish one other fundamental process. There has to be a mechanism that allows their control of the chromatin modification events that lead to gene transcription. There is evidence that binding site recognition by transcription activators is determined by chromatin context [33]. The previous knowledge of the importance of chromatin structure and function in the eukaryotic gene regulatory process is ancient and deep [12,34–37]. This is also true of the importance of the placement of linker histone H1 during gene transcription [38] and the effect of histone modifications in transcriptional regulation [39,40]. Willis suggested that the phosphorylation of linker histone H1 would be the crucial event leading to a change from heterochromatin to a less condensed state allowing access to consensus sequence-specific DNA binding sites by transcription activators such as vital oncogenes [12]. The subsequent confirmation of the effect of histone H1 phosphorylation on histone H1’s secondary structure and DNA condensation has well been demonstrated [41,42]. The relevance of such phosphorylation events cannot go unnoticed in the context of potential events during vital oncogene action within the nucleus.

If vital oncogenes are indeed the true wardens of carcinogenesis, achieving their goals by usurping cell cycle control mechanisms and intruding upon the normal mechanisms of gene transcription that leads to an addictive dependence by cancer cells, then they shall become the Achilles heel that we have long sought after. They should become the target for the development of molecularly targeted cancer therapy. Yet, before such an idealistic world can exist, we must first develop the tools and methodology needed to identify and isolate the oncogenic protein products of these entities. Although it is clearly recognized that cancer is a genetic disease [26], an even more appropriate perspective is that it is a disease of molecular dysfunction. This is so because the fact is that it is the molecular by-products of these oncogenes that are the true actors responsible for the seemingly demonic transformation of beautiful biochemical cascades into wayward pathways of independent self-indulgence and host death.

The final defeat of this disease will occur with the emergence of the practical applications of genomics and proteomics [43]. There can be no greater impact on translational medicine then the knowledge gained from the completion of the human genome project. The identification of the human genetic landscape has directly allowed the development of high-throughput technologies that include genetic mapping and identification of potential target sequences involved in the neoplastic process. The investigation of gene expression profiles using complementary DNA microarrays is rapidly dominating the initial efforts to understand critical alterations in the transcriptome that may reflect the distinct footprints of the consequences of the controlling effects of vital oncogenes [44,45]. But the supreme contribution this particular technology will play in our increasing knowledge of the cancer process will be the ability to directly identify the gene targets of transcription activating vital oncogenes such as c-*myc* [46].

The multifaceted aspect of the applications of protein microarray technology belies its inevitable incomprehensibly important role in the future design and development of truly personalized targeted cancer therapy [47]. Signal transduction is the chosen biological mode by which the human cell reacts to its microenvironment, regulates its critical biochemical pathways, and alters the activities of its genes [48]. Mass spectrometry proteomic techniques may be used to profile signaling proteins in cancer cells [49]. But it is the evolution of special analytical mass spectrometry proteomic techniques that will make the greatest contributions to the development of the ultimate specific diagnostic tests and targeted therapies that will define personalized cancer care. The significant role of phosphorylation reactions in normal human cell function is now self- evident. Protein kinases account for nearly 2% of the human genome [50]. And one-third of all proteins in the eukaryotic cell are phosphorylated at any given time [51]. A starting point is the analysis of the phosphoproteome in normal and cancer cells [52]. Surely the identification of distinct phosphoproteins with characteristic function-altering phosphorylation patterns will identify the important role that these vital oncoproteins are playing in the cancer process [53]. It can be assumed that antibody-based proteomics will be the corner stone of this effort [54]. Ultimately, both genomic and proteomic techniques will be utilized routinely in the future to decipher the molecular and pathophysiological characteristics of the individual cancer of an individual patient. This will be the essence of true personalized cancer care.

## 2. Gene Control by Phosphoproteins: A Basic Theory

Although gene regulation in humans is complex, and much has been learned in the past two decades, the elementary theoretical foundation for understanding normal and abnormal human genome function has been previously presented [12]. This theory proposed the following. First, human gene regulation requires a specific but simple general mechanism for gene activation. Second, gene activation should be achieved by a reversal of non-specific genomic suppressive processes. Third, both genomic structure and biochemical events are crucial to the activation and deactivation of genes. The specific and most important biochemical events are phosphorylation reactions. Fourth, oncogenes usurp and mimic the normal function of proto-oncogenes during carcinogenesis. Fifth, the existence of vital nuclear oncogenes is predictable. Sixth, the phosphorylation of both DNA and RNA polymerase not only plays an important role in normal gene regulation, but the phosphorylation of both of these molecules is facilitated during oncogene action. Seventh, dephosphorylating reactions and protein phosphatases play an important role in cell regulation by proto-oncogenes and cell deregulation by oncogenes, as a result, suppressor proteins with phosphatase activity must exist. Eight, there will exist a host of dysfunctional proto-oncogenes with protein kinase activity. Ninth, the mechanism for viral transformation of eukaryotic cells duplicates the same transformation process by oncogenes [55]. Tenth, steroid hormone receptors are phosphoproteins that function as nuclear transcription activators and regulate the human genome in the same manner as proto-oncogenes.

The suggested general events that lead to normal gene transcription were described as follows:

1. Nuclear proto-oncogene protein kinases are activated by phosphorylation.
2. These phosphorylated activated protein kinases bind to recognition sites in the major groove of the DNA helix as it winds around the nucleosome core histones.
3. A series of phosphorylation reactions mediated by these nuclear phosphoproteins and facilitated by histone H1 phosphorylation result in the displacement of histone H1 away from the site of gene activation eventually allowing access by the transcription initiation complex.
4. The exposed DNA sequences are essentially promoter sites.
5. The phosphorylation reaction cascade includes recruitment and phosphorylation of either DNA polymerase or RNA polymerase.
6. The intact histone octamer that makes up the nucleosome core complex functions as a scaffold during gene activity. The model assigned a central role to nuclear phosphoproteins.

We now know that phosphorylation reactions are the foundation of the signal transduction pathways that ultimately target transcription activators, their coregulators, and associated chromatin-modifying factors, leading to their phosphorylation by protein kinases or dephosphorylation by protein phosphatases [56,57]. We also now know that chromatin modifying events are essential for normal eukaryotic gene regulation. The debate continues, however, concerning the role that specific biochemical events such as acetylation and methylation must play in gene regulatory processes [58,59]. But a review of the literature overwhelmingly supports the pivotal role played by histone H1 phosphorylation in transcription and replication in both normal and transformed cells [60–62]. Only within the past ten years has sufficient data been generated verifying the yin and yang roles protein kinases and protein phosphatases play both in normal cell function and the cellular events transpiring during carcinogenesis [15,19]. Furthermore, distinct nuclear protein phosphatase inhibitors of nuclear located transcription activators have been identified [17]. Surprisingly, an interesting group of phosphatases have been identified which control the MAP kinase cascade. These are dual specificity phosphatases a subclass of the protein tyrosine phosphatase gene superfamily with the capacity to dephosphorylate the critical phosphothreonine and phosphotyrosine residues found among MAP kinases [18]. Only recently has evidence shown that protein tyrosine phosphatases can function as tumor suppressors [20]. A unique dual-specific phosphatase tumor suppressor gene has been identified [63].

The transforming mechanisms for DNA tumor viruses have become increasingly evident also only recently. As will be discussed later for vital oncogenes, tumor viruses with dominant acting oncoproteins exert their effects by interacting with key cellular targets resulting in the disruption of the normal controlling constraints placed on cell replication and gene transcription. The SV-40 large T antigen interacts with Rb, p53 as well as the pp2A phosphatase during transformation [64]. Likewise, research has revealed an interaction between SV-40 small T antigen and cyclin E resulting in an enhanced action of the cyclin-dependent kinase CDK2, a key regulator in the passage of the cell through the cell cycle restriction point between G1 and the DNA synthesizing S phase [65]. SV-40 small T antigen also has been shown to stimulate cyclin D1 promoter activity [66]. Cyclin D1 is the regulatory protein which cyclin-dependent kinases CDK4 and CDK3 depend upon for their phosphorylation-driven activation to also allow passage of the cell completely through the G1 phase and into the S phase.

Steroid hormone receptors and steroid hormone action have been the topic of intensive basic and clinical research for decades. This nuclear hormone receptor superfamily contains the receptors associated with not only steroid hormones, but thyroid, vitamin D, and retinoid hormones as well. Many are ligand associated regulatory proteins. In fact, it is now clear that they all function as nuclear receptors by directly interacting within the region of the DNA response elements of target genes. Among other events, we now know that some hormone receptors have the capacity to recruit coregulators and interact by cross-talking to other signaling pathways. Even more revealing is the presence of some of the unique characteristics we now typically attribute to the cell surface protein kinase receptors involved in signal transduction during uncontrolled cell proliferation. For example, nuclear hormone receptors undergo a conformational change following ligand banding. The activation process of nuclear hormone receptors also often involves a formation of homodimers or heterodimers, the functional molecule. Of note, the recruitment of coactivator complexes to the target promoter during gene transcription results in chromatin decompaction [67]. Steroid hormone receptor phosphorylation is now well demonstrated and understood. Moreover, the exquisite details of the physiologic dynamics of steroid receptor phosphorylation have become increasingly clear over the past few years [68]. It appears that the phosphorylation of the steroid receptor modulates a host of its functions, including its stability, nuclear localization, hormone sensitivity, DNA binding, and protein-protein interactions that determine the specificity and extent of the regulation of target genes [69]. In addition, unliganded estrogen receptor mutants may function as downstream targets of the MAP kinase transduction pathway resulting in their direct phosphorylation [70]. The implication of this as a molecular explanation for the particular virulence of estrogen resistant breast cancer is obvious. A yin and yang relationship is also evident in the relationship between estrogen receptors and protein phosphatases. The transcriptional activity of the estrogen receptor is halted by the dephosphorylating inhibitory effect of a protein phosphatase [71]. Finally, steroid receptor action is mediated by one of the same mechanisms proposed here for vital oncogenes. Cyclin-dependent kinases activity is needed as a coactivator [72]. All of the subsequent research supports the original concept that steroid receptors function like proto-oncogenes in normal cells. It therefore is further hypothesized that the molecular basis for the virulence of estrogen receptor negative breast cancer is a mutational change in the receptor affecting critical phosphorylation sites that result in hormone independence and the acquisition of the usurping characteristics of a vital oncogene.

The continued validity of this phosphoprotein-driven model of the regulation of the eukaryotic genome should be based on the judgment of its relevance to what is currently known about the detailed biochemical and molecular aspects of human gene regulation, its testability, its compatibility with current knowledge, its predictive power, and its level of simplicity.

**Figure 1:**
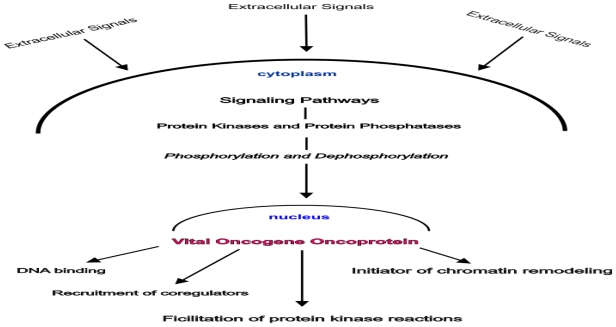
Biochemical mechanisms in vital oncogene action. The oncoprotein products of vital oncogenes are the ultimate end targets of the cancer cell’s signaling pathways. Their cellular activities occur within the nucleus.

“A theory is a good theory if it satisfies two requirements: It must accurately describe a large class of observations on the basis of a model that contains only a few arbitrary elements, and it must make definite predictions about the results of future observations [73].”(Figure 1)

## 3. The Role of Cyclin-Dependent Kinases and the pRB Protein in Cell Cycle Control

**Figure 2:**
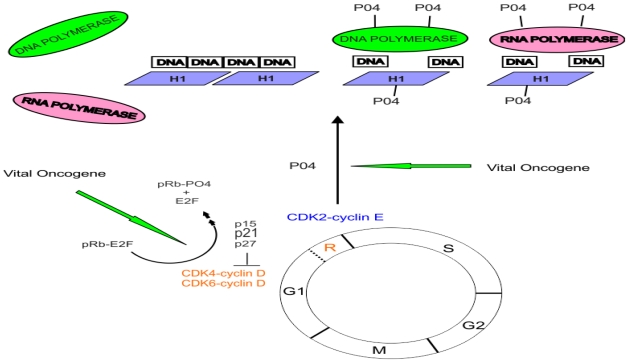
Usurpation of cell cycle control and transcription by vital oncoproteins. The oncoprotein products of vital oncogenes could enhance the cyclin-dependent kinase phosphorylation activities resulting in phosphorylation of the Rb suppressor protein and histone H1.

A defect in the normal cell cycle control mechanisms is an essential component of carcinogenesis [74]. The normal eukaryotic cell cycle consists of a series of well-orchestrated events ending in the production of two daughter cells. The stages of the eukaryotic cell cycle have been known for some time [75]. The cell cycle is composed of four phases (Figure 2). The entire process consists of two critical steps. During the S phase DNA is replicated. This ultimately leads to the M phase during which time cell mitosis occurs allowing the separation of duplicated chromosomes. The other two phases consist of gap periods. During G1 the cell prepares all of the necessary machinery needed for DNA replication. Following DNA replication a second pause occurs. It is during this second gap (G2) that the cell now prepares all the necessary machinery for cell division. But not all cells continue through this process. Some sidestep into a resting or quiescent phase designated G0 where they may remain in a fully differentiated state. Cells may exit this state and re-enter the cell cycle as a result of internal or external stimulating events. It is crucial that the processes that drive the cell through its cell cycle are securely and efficiently regulated. The heart of this time-controlled mechanism lies at designated stop checkpoints that are the gatekeepers for the transitions between the phases of the cell cycle. These are the restriction points controlled by a family of protein kinases designated cyclin-dependent kinases, CDKs [76–78]. Cyclin-dependent kinases CDK4 and CDK6 regulate passage of the cell cycle through the restriction check-point (R) placed between the G1 and S interface. Cyclin-dependent kinase CDK2 later facilitates the transition process (Figure 2). If the R checkpoint is usurped, it follows that the cell may inappropriately proceed through the cell cycle repeatedly. This is the classical cellular behavior found in cancer.

At least 11 mammalian CDKs have been identified [77]. They are the key regulators of the cell cycle [79]. These small molecules consist of little more than the catalytic core common to all protein kinases. Their enzymatic activation is completely dependent upon the binding of a regulatory cyclin subunit, but full activation requires the phosphorylation of a threonine residue residing near the CDK’s active site. Several regulatory cyclin proteins have been identified. Cyclin D is specifically associated with CDK4/6 while cyclin E regulates CDK2. During the cell cycle, the levels of the cyclin regulatory proteins rise and fall at the appropriate time while the levels of the CDKs remain the same. CDK7 plays a distinct role. It is designated as a CDK-activating kinase (CAK) that phosphorylates other CDKs [80,81]. Because of this vital role, it follows that any event that could usurp this protein’s function in the cell cycle could result in the command of a crucial process in cell cycle regulation. Cyclin-activating kinase CDK7 therefore potentially regulates all of the phosphorylation activities of CDK4/6 and CDK2. The essential roles of CDK4/6 and CDK2 are to control cell cycle progression through phosphorylation of proteins that function at specific cell cycle stages. This includes the protein produced by the retinoblastoma tumor suppressor gene, pRb [82]. The CDKs in turn are regulated by a host of inhibitory proteins. Some of these cyclin-dependent kinase inhibitors interact with the CDK4/6 complex to block kinase activity during G1 [83,84]. Deregulation of the normal functions of CDK and cyclin is associated with the development of cancers in humans [85–93].

The pRb tumor suppressor protein is the master break shoe preventing the progression of the cell completely through the G1 phase of the growth cycle and into the S phase [94,95] (Figure 2). Only the unphosphorylated Rb protein has this functional capacity. It achieves this by binding to E2F transcription factors. The binding of the unphosphorylated Rb protein to E2F halts the binding of E2F to not only its consensus promoters, but also the promoters of such proto-oncogenes as c-*myc* and c-*fos.* E2F in turn activates the expression of cyclin E and CDK2 which normally further promotes the progression of the cell through the latter part of the G1 phase and well into the S phase. It is the state of the phosphorylation of pRb that determines its affinity for E2F. This affinity is greatest when pRb is hypophosphorylated. As expected, phosphorylation of pRb is maximal at the start of the S phase and lowest after mitosis. Furthermore, the phosphorylated Rb protein has the capacity to stimulate DNA polymerase alpha [96]. A loss of normal pRb function means the loss of control over the cell cycle.

One more crucial interaction remains. In the normal cell cycle both CDK2 and CDK 4/6 phosphorylate pRb which eliminates its inhibitory function on E2F and enhances its ability to stimulate DNA polymerase [96–98]. The overall processes of normal cell cycle control can now be summarized from these facts. Cyclin dependent kinases 2 and 4/6 are the proteins of interest here. They control cell cycle progression by phosphorylating the retinoblastoma tumor suppressor protein. The Rb protein now in a phosphorylated state loses its inhibitory binding capacity over E2F. The E2F-Rb protein complex dissociates releasing the two active components—an active transcription factor, E2F that promotes expression of cell-proliferation genes, and a phosphorylated Rb protein that stimulates DNA polymerase alpha leading to DNA replication (Figure 2). There are several superb reviews of these concepts [28,74,94,99].

## 4. Genomic Structure and Epigenetic Regulation

Unlike within the prokaryotic cell, within the eukaryotic cell the genome has bulk. It has structure. It has contour and conformation. This characteristic arises as a result of the presence of chromosomal proteins [34,36,37]. Within this milieu resides an assortment of actors, including general transcription factors, DNA and RNA polymerases, and recruited chromosomal protein modifying enzymes. But our focus here will be on the role of the histone complex—especially histone H1.

The compacted structure of heterochromatin and the loose open structure of euchromatin have been repeatedly studied, as well as the dynamic transformation of closed compacted DNA to a more open accessible state during transcription and DNA replication [35,100]. Although it is quite clear that the octamer nucleosome core complex serves as a scaffold during this process, the dynamic and specific role of histone H1 along with the post-translational modifications of the histone complex are still under intense study. Accumulated data continues to reveal the two functional aspects of chromatin modification which include effects that directly alter chromatin packing perhaps by a change in electrostatic charge, or an alteration of the nucleosome surface characteristics and DNA contour which could promote the recruitment of effector protein complexes. The latter phenomenon suggests that the creation of “epigenetic” marks could serve as perhaps signposts recognized by transcription activators and the associated effector protein complex [33,40,59]. Not only do modifications of the histone proteins play a role during transcription, but also the alterations of DNA as well. The relevance of the status of DNA methylation is evident in the altered patterns of DNA methylation found in neoplasia [58]. But it is proposed here that aberrant alterations of the DNA methylation patterns seen in neoplasia are not a reflection of the specific mechanism for the regulation of eukaryotic genes [101]. Altered patterns of DNA methylation seen in neoplasia more likely are a reflection of the methylation alterations induced in the setting of gene control gone wild as a direct result of the activity of a vital oncogene during its usurpation. In essence, hypermethylation and the inappropriate transcriptional silencing of genes is a response to oncogene activity. This “shutting-down” of genes probably includes especially tumor suppressor genes. It is a way by which vital oncogenes may suppress the suppressors.

The phosphorylation of linker histone H1 achieves the regulation of gene expression by mimicking H1 removal from compacted chromatin, resulting in decondensation and promoter gene exposure to the transcriptional complex [41,42]. Furthermore, site-specific interphase H1 phosphorylation facilitates transcription by RNA polymerases I and II [102] (Figure 2). It also appears that H1 phosphorylation regulates the ATP-dependent chromatin remodeling enzymes in a global fashion [103]. The cell cycle control protein CDK2 in turn phosphorylates histone H1 [104]. We can now visualize the completion of the cell growth cycle starting with the activation of the CDKs, followed by deactivation of pRb, then phosphorylation of histone H1, with subsequent decondensation of the chromatin complex, and finally phosphorylation-mediated activation of polymerases. As expected, the phosphorylation of histone H1 plays a vital role in carcinogenesis including transformation caused by c-myc [60–62].

## 5. c-*Myc* a Prototype Vital Oncogene

A vital oncogene must have certain fundamental characteristics. It must have a proven association with cancer. Its direct effect must occur within the nucleus. Its oncoprotein product must be a downstream target for any associated initiating protein kinase cascade. Its oncoprotein product must usurp or alter normal cell cycle control mechanisms. Finally, there should be clinical evidence of oncogene addiction. c-*My*c fulfills these criteria. The capacity of overexpressed c-*myc* to initiate and facilitate proliferation, and its association with diverse cancers is incontrovertible [105–107]. Accumulated research over the prior decade has increasingly confirmed the effect of c-myc on cell cycle regulation. It appears that c-myc has an effect on the frequency of initiation of the cell cycle. It affects the decision of cells to enter or exit the cell cycle [108,109]. Repression of c-myc in cycling cells causes arrest in G1 as a result of the reduction of cyclin E/CDK2 kinase activity [110]. Additional evidence has revealed the relevant genomic targets of the c-Myc oncoprotein [111]. Most revealing, however, is the differential genetic modulation and activation of CDK4/6, CDK2 and associated cyclin proteins by c-Myc [112–115]. Finally, the c-Myc oncoprotein directly interacts with the Rb suppressor protein [116]. The cell cycle effect of other potential vital oncogenes has been demonstrated as well [117,118].

Although c-*myc* effects, no doubt, are mediated to some extent through direct gene activation of cell regulatory target genes we cannot exclude a direct effect of the c-Myc oncoprotein on the cell cycle control cyclin dependent kinases and pRb. The binding of Tax oncoprotein of the human T-cell leukemia virus type I to CDK4 directly stimulates the phosphorylation of pRb [119]. Direct phosphorylation of CDK4 by the Src family of kinases occurs [120]. Here, it is proposed that vital oncogenes function by facilitating the phosphorylation of vital proteins involved in human gene regulation. These include the cyclin dependent kinases CDK 4/6 and CDK2, the Rb suppressor protein, histone H1 and the DNA and RNA polymerases (Figure 2).

## 6. Conclusion

There has been a less than silent revolution in cancer care for more than sixty years, starting with the initial growth factor research by Levi-Montalcini and Cohen [121]. This work has led directly to the identification of the functional characteristics of cell growth factor receptors and their associated receptor protein kinase cascades, as well as to the discovery of therapeutic protein kinase inhibitors [122–125]. This is the evolution of truly targeted cancer therapy. Phenomenal clinical results have been achieved in lung cancer and melanoma therapy [126,127]. The next revolution will be in the personalization of cancer care directed by utilization of targeted molecular therapies. Thus far the term targeted therapy has been applied to drugs that are designed to inhibit predominantly signal transduction pathways thought to be critical for cancer cell growth and survival. However, there is a shortcoming to this approach. The difficulty results from our inability to identify which molecular target in any given cascade is the truly sentinel entity standing as the most important effector of the neoplastic process. This difficulty is evident when we look more closely at the overall clinical benefit of our current biologically targeted cancer therapies [128]. Ideal cancer targets must be molecules that are vital to the malignant phenotype. These entities must not be significantly expressed in a normal fashion in all other tissues. Aberrantly functioning transcription activators could fulfill this role [129]. There are considerably more human oncogenes in signaling pathways than oncogenic transcription activators. Still, obviously, there has to be a selective disruption of the transcription process. Here, it is proposed that vital oncogenes are rational targets for achieving personalized cancer care. The next great obstacle is how to go about identifying such entities.

Cancer results from a deranged genome manifested by the commonly seen associated genetic defects, and evident in the resulting dysfunctional protein products of these defective genes that drive the cancer process. Advanced diagnostic techniques capable of detecting and reading genomic and proteomic information in an efficient, sensitive, and specific way will be the foundation upon which individualized cancer treatment will stand. Two evolving technologies will fulfill these criteria [43]. Genome-wide association studies of cancer have identified many important regions of genetic variation associated with cancer [130,131]. The technique of DNA microarray analysis, a high-throughput Southern blot technique, allows comparative assessment of genomic mutations, polymorphisms, and epigenetic alterations [45]. Analysis of gene expression profiles may help identify a host of potential vital oncogenes [132,133]. This investigative approach may help to identify not only new genome associated cancer markers, but will lead directly to development of specific drugs targeting the gene products of these gene candidates [134]. As this technology evolves, it may achieve the capacity to effectively identify specific vital-oncogene associated gene expression patterns. Therefore, gene expression signature evaluations by DNA microarray techniques will allow a new way of molecularly classifying cancers, adding important information needed to determine the probability that a specific cancer will respond to a specific therapy directed against the vital oncogene driving the gene expression pattern seen in that particular cancer [135–137].

**Figure 3:**
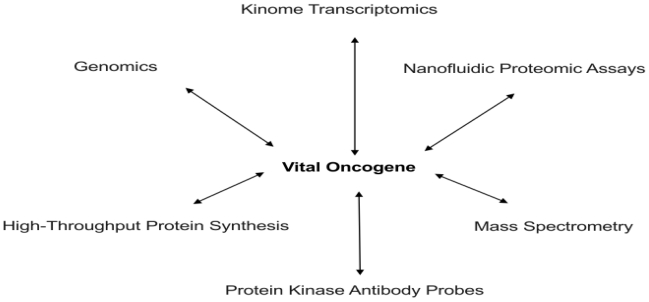
Diagnostic approaches used to evaluate vital oncogene action.

There also exists another potential footprint of vital oncogene activity. This will be evident within the aberrant and specific protein patterns detected by proteomic techniques [47]. Although mass spectrometry is too crude for the ultimate specificity desired, even this approach reveals potential tumor associated signaling proteins in cancer cell lines [49]. More relevant is the capacity to demonstrate a cancer’s dependence upon a specific transduction pathway [138]. Even more exciting is the ability to potentially identify a vital oncogene by comparative proteomic analysis [139]. Yet, even this is insufficient. We desire a specific, clinically feasible, and applicable approach that will identify the specific gene product of a vital oncogene. That specificity can be achieved by phosphoproteomic techniques, particularly utilizing antibody based methods [53,54,140]. Ultimately, however, nanofluidic proteomic assay analysis which is based upon the effect that post-translational modifications have on a protein’s isoelectric point will supersede all of these efforts. This method allows quantitation of low-abundance protein isoforms in nanoliter volumes. For example, quantitation of the vital oncoproteins produced by the vital oncogenes c-*myc*, *stat-3*, and c-*jun* has been demonstrated [141]. Figure 3 summarizes the diagnostic approaches that will serve as the foundation for the development of truly individualized cancer therapy.

## Supplementary Material
