# Supplementary figures and images for: Human Gene Control by Vital Oncogenes: Revisiting a Theoretical Model and Its Implications for Targeted Cancer Therapy
Source: Int J Mol Sci. 2011 Dec 27;13(1):316–35. doi: 10.3390/ijms13010316 (PMC3269688; doi:10.3390/ijms13010316)

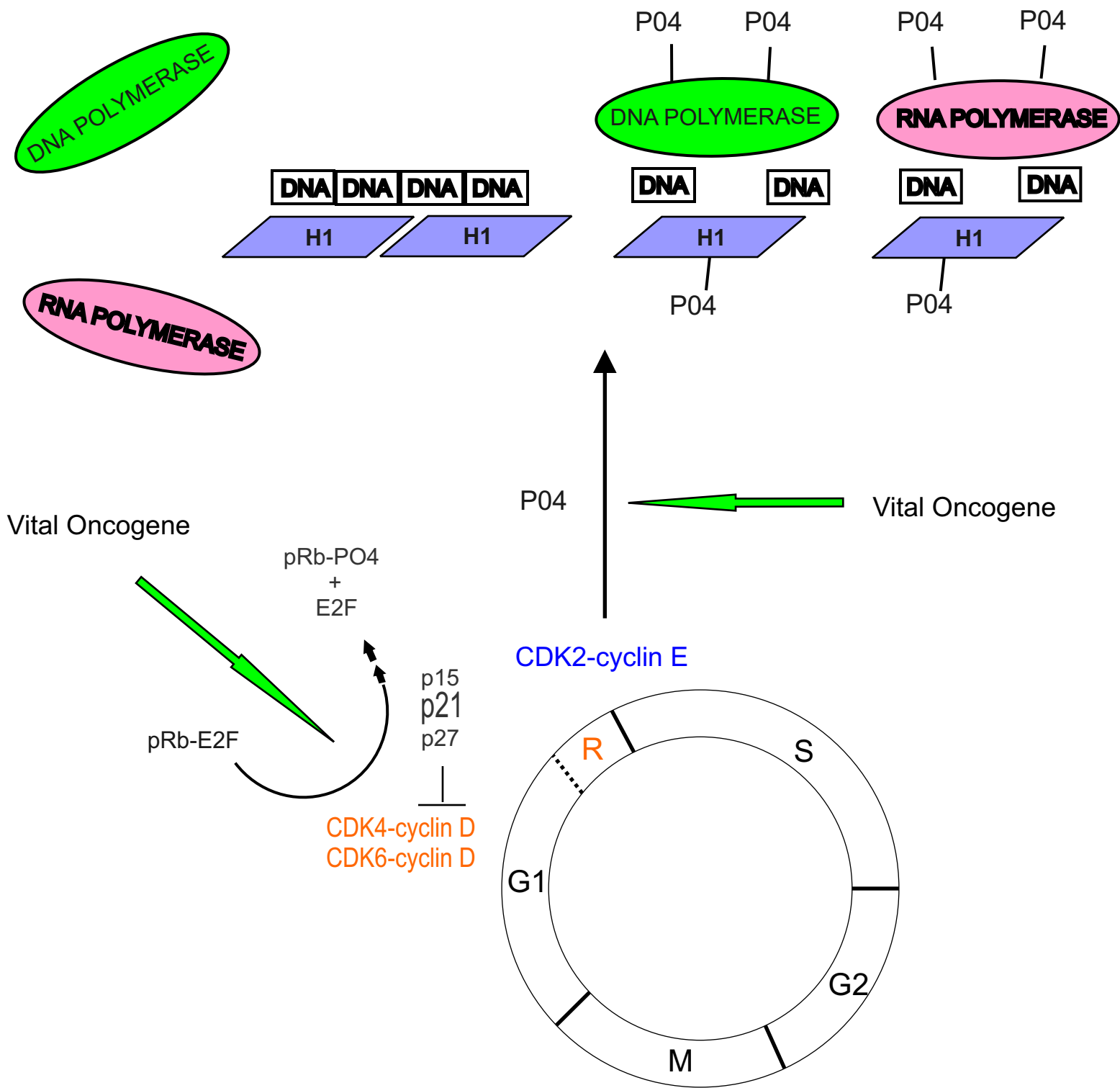

Figure 2

Supplement: Supplementary file 1 [file ijms-13-00316-s001.pdf]
